# *Brassica* biodiversity conservation: prevailing constraints and future avenues for sustainable distribution of plant genetic resources

**DOI:** 10.3389/fpls.2023.1220134

**Published:** 2023-07-27

**Authors:** Parthiban Subramanian, Seong-Hoon Kim, Bum-Soo Hahn

**Affiliations:** National Agrobiodiversity Center, National Institute of Agricultural Sciences, Rural Development Administration, Jeonju-si, Jeollabuk-do, Republic of Korea

**Keywords:** plant genetic resources, genebank, *Brassica* biodiversity, taxonomy, *Brassica* conservation, *Brassica* species

## Abstract

The past decade has seen an observable loss of plant biodiversity which can be attributed to changing climate conditions, destroying ecosystems to create farmlands and continuous selective breeding for limited traits. This loss of biodiversity poses a significant bottleneck to plant biologists across the globe working on sustainable solutions to address the current barriers of agricultural productivity. Plant genetic resources centers or genebanks that conserve plant germplasm can majorly contribute towards addressing this problem. Second only to soybean, *Brassica* remains the largest oil-seed crop and is cultivated across 124 countries, and FAO estimates for a combined gross production values of broccoli, cabbages, cauliflower, mustard and rape seeds stands at a staggering 67.5 billion US dollars during the year 2020. With such a global status, wide variety of uses and more recently, growing importance in the health food sector, the conservation of diverse genetic resources of *Brassica* appeals for higher priority. Here we review the current status of *Brassica* conservation across plant genebanks. At present, at least 81,752 accessions of *Brassica* are recorded to be conserved in 148 holding institutes spread across only 81 countries. Several aspects that need to be addressed to improve proper conservation of the *Brassica* diversity was well as dissemination of germplasm are discussed. Primarily, the number of accessions conserved across countries and the diversity of *Brassica* taxa most countries has been highly limited which may lead to biodiversity loss in the longer run. Moreover, several practical challenges in *Brassica* germplasm conservation especially with respect to taxonomic authorities have been discussed. The current review identifies and highlights areas for progress in *Brassica* conservation, which include but are not limited to, distribution of conserved *Brassica* biodiversity, challenges faced by conservation biologists, conservation methods, technical hurdles and future avenues for research in diverse *Brassica* species.

## 1 Introduction

*Brassica* is one of the highly diverse and largest genera of plants, cultivated and consumed all over the world in its different forms (Fahey, 2016; El-Esawi, 2017). Members of the genus *Brassica* include, but are not limited to morphologically diverse crops including bok choy, broccoli, Brussel sprouts, cabbage, cauliflower, canola, Chinese cabbage, kale, kohlrabi, mustard, rapeseed, and turnips. These plants, given their enormity in diversity and distribution, have equally substantial uses to mankind both directly as health foods, in cuisines, source of oil, source of therapeutics and indirectly as energy crops, in pest control as well as biofumigants (Dixon, 2006; Cornblatt et al., 2007; Szczygłowska et al., 2011; Fahey, 2016; Hagos et al., 2020; Wijaya et al., 2020). Therefore, it is important to study the diverse *Brassica* species to tap their genetic potential for making the best use of them in the future. To accelerate worldwide research on *Brassica*, conservation and dissemination of *Brassica* genetic resources becomes essential with the responsibility falling on plant genetic resource centers or plant genebanks to ensure adequate supply of germplasm. Also, given the narrowing gene pool in *Brassica* crops as a result of continuous breeding for limited selective traits and overall loss of biodiversity due to changing climate conditions, genebanks offer a great choice for conservation of its plant genetic resources including cultivars, crop wild relatives (CWR) and landraces. The International Treaty on Plant Genetic Resources for Food and Agriculture (Plant Treaty) was framed in 2001 for sustainable use of the plant genetic resources for food and agriculture (PGRFA) across the world (Panis et al., 2020; Pathirana and Carimi, 2022). Currently, the Consortium of International Agricultural Research Centers (CGIAR) maintains a network among 15 research centers and with a presence in 89 countries conserves more than 770,000 accessions of plants (CGIAR, 2022). On the other hand, the WIEWS (World Information and Early Warning System) of FAO connects 22,708 member institutes spread across 114 countries that conserve 5.7 million accessions of plant species (WIEWS, 2022). Both of these institutions strive conservation and adequate supply of the plant genetic resources (PGRs) to the global community.

With regards to conservation of PGRs, continuous research is being carried out to 1. Optimize the storage conditions for different crop germplasm as several factors influence the quality of conserved germplasm during long-term storage; 2. Improve the data availability and networking by optimizing the current gene bank information management systems (Kameswara Rao et al., 2017; Weise et al., 2020). This has led to adequate resources being published for proper documentation as well as conservation of PGRs in genebanks (Reed et al., 2004; Food and Agriculture Organization of the United Nations, 2013; Kameswara Rao et al., 2017; Langridge and Waugh, 2019; Volk et al., 2019). In the present agricultural scenario, the responsibility of conserving the genetic diversity of specific plant species and distribution to breeders as well as plant researchers fall majorly on the genebanks. In this present review, we try to summarize the current status of *Brassica* conservation at genebanks in terms of availability and diversity, the challenges faced by the genebanks in *Brassica* germplasm, hurdles in field collections, and future need for conservation of *Brassica* biodiversity.

## 2 Current status of *Brassica* conservation

### 2.1 Data collection

To collect updated information on Brassica genetic resources, the following webpages were accessed: FAO-WIEWS, ITIS (Integrated Taxonomic Information System), POWO (Plants of the world online), Genesys, EURISCO, GRIN (Germplasm Resources Information Network), GBIS-IPK (Genebank Information System of the IPK - Institut Für Pflanzengenetik Und Kulturpflanzenforschung Gatersleben), UKVGB (United Kingdom Vegetable Gene bank), RDA (Rural Development Administration, S. Korea), NARO (National Agriculture and Food Research Organization), VIR (N.I. Vavilov All-Russian Scientific Research Institute of Plant Industry), WVC (World Vegetable Center), and CGN (Centre for Genetic Resources, the Netherlands) were accessed. For literature review the keywords used were “*Brassica* germplasm”, “*Brassica* conservation”, “Plant genetic resources”, “*Brassica* biodiversity” at Google scholar website (www.scholar.google.com). Only publications that discussed *Brassica* physiology or germplasm conservation strategies and/or policies were chosen. Preference was given to publications on policies and germplasms status updates published after the year 2017.

### 2.2 Plant genetic resource centers and *Brassica* germplasm collections

**Figure 1:**
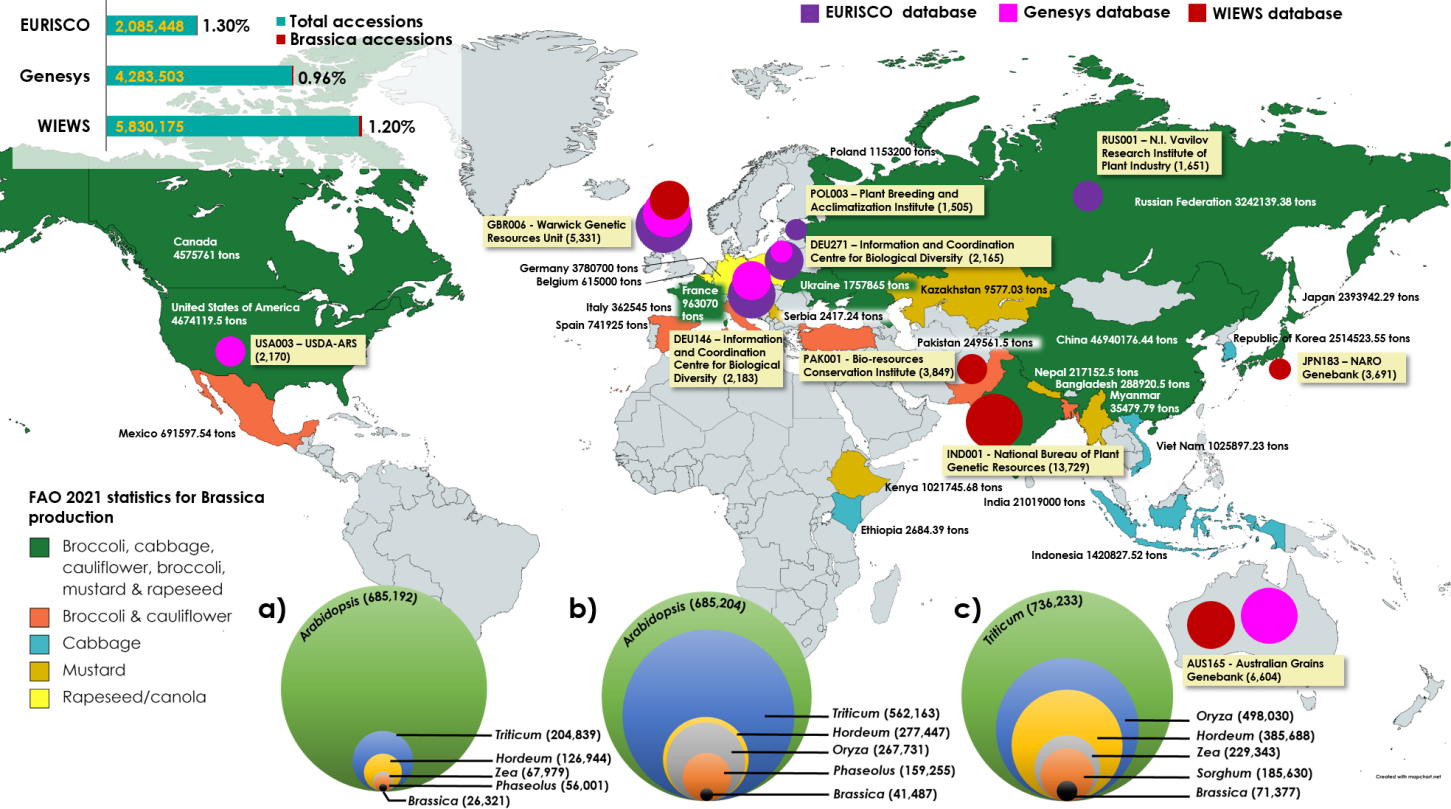
*Brassica* production statistics (FAOSTAT) during the year 2021 and top five *Brassica* plant genetic resource holding centers according to FAO-WIEWS, Genesys and EURISCO databases. Labels contain the institutional code followed by the name of the organization and number of Brassica accessions held (in parentheses). Highest plant germplasms conserved according to FAO-WIEWS, Genesys and EURISCO databases (a, b & c) respectively. The world map in the background was created at www.mapchart.net under Creative Commons Attribution-ShareAlike 4.0 International License which permits it to be free to copy and redistribute.

While WIEWS remains to be the global platform for information on conserved plant germplasm, two online data repositories, Genesys, managed by the Crop Trust and the European Search Catalogue for Plant Genetic Resources (EURISCO) also cater as major online directories for obtaining ordering information on specific accessions. Genesys database provides information on about 4.1 million accessions conserved in 450 genebanks all over the world of which approximately less than 1% (0.96%, 41,487 accessions as on 1^st^ December, 2022) belong to *Brassica* (Genesys, 2017) (**Figure 1**). EURISCO stores data of about 2 million accessions of plants and their wild relatives, preserved in 400 institutes located in 43 member countries of the European Cooperative Programme for Plant Genetic Resources (ECPGR) (Weise et al., 2017). EURISCO stores only 26,321 accessions of *Brassica* (as on 1^st^ December, 2022) which constitute to only 1.3% of the total accessions in the database (**Figure 1**). *Brassica* also does not appear among the list of top ten crops with at least 100,000 accessions conserved worldwide (Börner and Khlestkina, 2019). However, European nations are major contributors to the overall production of Broccoli, cauliflower and rapeseed/canola in the world with a combined output of over 7.6 million tons of agricultural crop production in 2021 (**Figure 1**) (FAOSTAT, 1997). There is an evident underrepresentation of *Brassica* accessions among the conserved germplasm despite their nature of being such a diverse and anthropologically important genus of crop plants (Kellingray et al., 2017; Wijaya et al., 2020; Salehi et al., 2021).

A comparison of overall *Brassica* production in 2021 and major plant genetic resources centers involved in conserving *Brassica* biodiversity across the world indicate that Asia, the United States and Europe remain to be major producers of *Brassica* crops (**Figure 1**). The WIEWS official data from the SDG 2.5.1.a indicates that a total of 71,378 accessions of *Brassica* are currently maintained *ex situ* all over the world (**Table S1**). Primary observation may indicate that the highest number of accessions being conserved in genebanks in Europe and Asia, but in terms of species diversity, after Europe, North America (USDA-ARS) conserves diverse species of *Brassica* germplasm (**Table S1**). Further analysis indicate that less than 10 accessions are conserved in genebanks from Africa (Niger, Nigeria, Eswatini, South Africa, Libya), Middle East (Cyprus, Tajikistan, Jordan, Lebanon), Eastern Europe (Georgia, Montenegro, Armenia) and South America (Argentina, Mexico) (WIEWS, 2022). However, these parts of the world also contribute significantly to the world *Brassica* production and fall among the top 5 producers of crops such as Broccoli, cauliflower, cabbage and mustard seeds (**Figure 1**). Improving the available *Brassica* species biodiversity and dissemination could considerably advance further breeding and research in these parts of the world.

**Figure 2:**
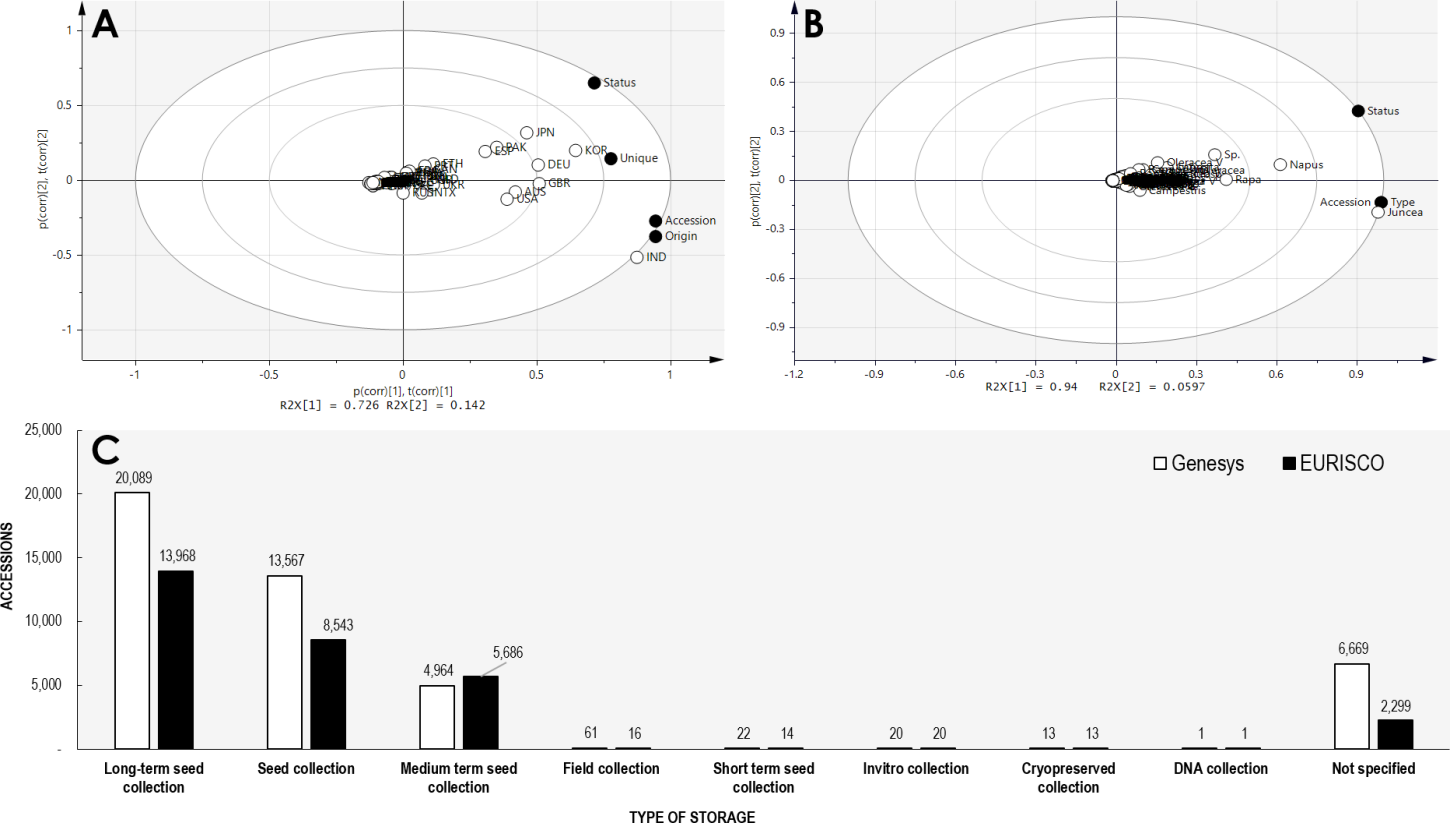
Principal component analysis of *Brassica* germplasm accessions. **(A)** Comparison of conserved *Brassica* accession by countries (accession – total accessions; unique – unique taxon of *Brassica*; origin – accessions with origin data available; status – accession with their biological status available). **(B)** Comparison of conserved *Brassica* accessions by taxon (accession – total accessions; status – accession with their biological status available; type – type of storage- long or short term data available). **(C)** Type of germplasm storage of *Brassica* germplasm in plant genebanks associated with online directories GENESYS and EURISCO (as accessed on 1^st^ December, 2022).

Currently, for ordering *Brassica* germplasm, the Germplasm Resources Information Network (GRIN) and Gene bank Information System (GBIS) from the US (maintained by Unites States Department of Agriculture - Agricultural Research Service (USDA-ARS) and Leibniz Institute of Plant Genetics and Crop Plant Research serve as channels where one can directly request *Brassica* germplasm for research purposes (Postman et al., 2010; Oppermann et al., 2015). In addition to these widely used genebanks, several other facilities are also available to source *Brassica* germplasm locally and internationally albeit the diversity being very limited (Liu et al., 2020). The current status of *Brassica* germplasm in major genebanks are given in the table below (**Table 1**). A Principal Component Analysis (PCA) of the available *Brassica* germplasm information indicated that country such as India conserves very high number of accessions (accession) whereas Korea, Germany, Great Britain conserves high diversity of *Brassica* species (unique). At the same time, high number of accessions conserved in Korea and Japan possess data on their biological status (status) (**Figure 2A**). Comparison of species indicates that *B. juncea*, remains the highly conserved *Brassica* taxon followed by *B. napus, B. rapa* and unsegregated B. spp contribute to the highest number of accessions stored in genebanks (**Figure 2B**). A majority of these *Brassica* germplasm are maintained as *ex situ* collections by germplasm collections and materials are preserved in short, medium and long term storage and available as seeds, plant material, and/or DNA (Walters et al., 2004; Acker et al., 2017; Lusty et al., 2021). A search on Genesys and EURISCO webpages indicate that a majority of *Brassica* germplasm are stored as long term storage, followed by seed collections and mid-term storage (**Figure 2C**). The major plant material conserved in all these three strategies are of seed type and seeds are the easiest way for exchange or dissemination of *Brassica* germplasm.

**Table 1:** Major genebanks and germplasm information (as accessed on 5^th^ October, 2022).

Country	INDIA	AUSTRALIA	KOREA	UNITED KINGDOM	RUSSIA	GERMANY	PAKISTAN	USA	JAPAN
Resource	NBPGR	AGG	RDA	UKVGB	VIR	GBIS-IPK	BCI	GRIN	NARO
*Brassica* accessions	13,729	6,604	6,399	5,314	4,881	4,351	3,849	3,167	1,833
Total accessions	420,324	164,044	272,351	12,158	243,829	129,748	23,722	42,743	225,811
Percentage *Brassica* germplams	3.27%	4.03%	2.40%	43.70%	2%	3.40%	16.23%	7.40%	0.80%
Species diversity	13	26	20	23	15	31	9	24	7
Subtaxa diversity*	42	43	58	29	41	77	12	63	25
Major taxa conserved	*B. juncea, B.campestris, B. rapa*	*B. rapa, B.napus, B. juncea*	*B. napus; B. juncea; B. rapa*	*B. oleracea; B. napus;* *B. rapa*	*B. juncea; B. oleracea; B. rapa*	*B. oleracea; B. napus; B. rapa*	*B.juncea, B. campestris, B. rapa*	*B. oleracea; B. rapa; B. napus*	*B. napus; B. rapa; B. oleracea*

NBPGR, National Bureau of Plant Genetic Resources; AGG, Australian Grains Genebank; RDA, Rural Development Administration; UKVGB, United Kingdom Vegetable Gene bank; VIR, N.I. Vavilov All-Russian Scientific Research Institute of Plant Industry; GBIS-IPK, Gene bank Information System of the IPK Gatersleben; BCI, Bio-resources Conservation Institute; GRIN, Germplasm Resources Information Network; NARO, National Agriculture and Food Research Organization. *Number of unique species, subspecies or varieties available.

### 2.3 Worldwide assessment of conserved *Brassica* germplasm

**Figure 3:**
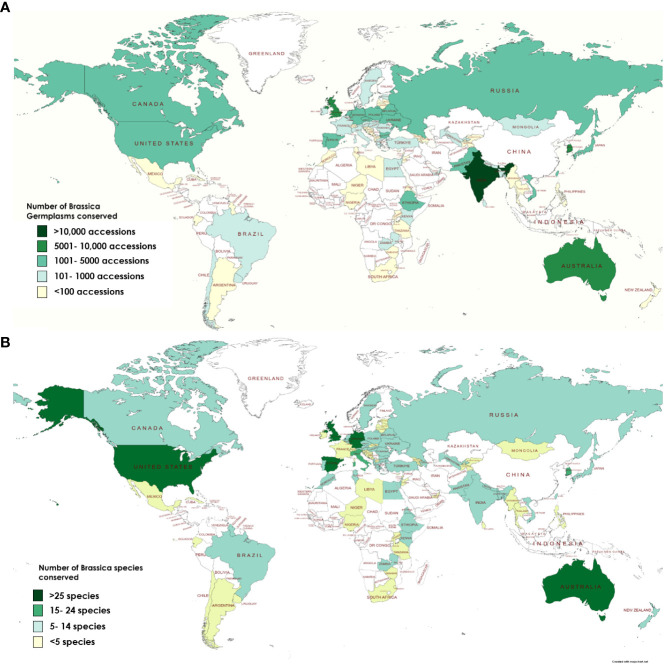
Conservation of *Brassica* biodiversity in terms of **(A)** Number of accessions stored **(B)** Species diversity across plant genebanks around the world.

Currently, comparison of the three online directories yields a total of 81,752 *Brassica* accessions stored in 81 countries across the world in addition to 3 regional and 3 international research centers (data summarized from WIEWS, Genesys & EURISCO). Additionally, few genebanks such as The National Crop Genebank of China (NCGC) and National Agrobiodiversity Center-RDA, South Korea also conserve *Brassica* germplasm but are limited for sharing of resources due to governmental policies. Historically, the six most common species of *Brassica* explained by the U triangle are well documented and are widely conserved all across the world (U, 1935) (**Figure S1**). It can be observed that genebanks that conserve high number of accessions of species such as *Brassica napus*, *B. oleracea* and *B. carinata* are located in vicinity to their geographical origins (**Figure S2**). Across Asia, green leafy vegetable crops of *B. rapa* (subsp. *chinensis* and *pekinensis*), oil producing rape *B. napus* and mustards of *B. juncea* with local origins are highly conserved in genebanks of India, Japan, Pakistan, Taiwan and South Korea (**Figure S2**). Co-ordination and dissemination of *Brassica* germplasm have resulted in countries such as Australia, Canada and the United States of America (USA) which are geographically far from the natural areas of origin of *Brassica* species to accumulate in both number and diversity (**Table 1**; **Figure 3**).

A country wise comparison of the WIEWS and Genesys databases for conserved *Brassica* accessions indicate that Australia, India, Germany, United Kingdom (UK), USA and Taiwan conserve the largest number of *Brassica* germplasm (**Figure 3A**). In terms of species diversity, the most common *Brassica* crop seeds conserved are *B. oleracea* (WIEWS: 17,389 accessions; Genesys 14,798 accessions); *B. rapa* (WIEWS: 15,132 accessions; Genesys 8,991 accessions) and *B. juncea* (WIEWS: 14,810 accessions; Genesys 4,698 accessions). However other *Brassica* species such *as B. tyrrhena, B. taurica, B. bivoniana, B. atlantica, B. nivalis*, and *B. aucheri* etc. are not highly conserved (<10 accessions). Moreover, there also exists disproportion in the biodiversity conserved at the plant genebanks. Among 146 plant genebanks recorded to conserve *Brassica >*38% geenbanks (56) have a *Brassica* biodiversity of less than 10 taxons (including subspecies and varieties) and largely conserve *B. oleracea, B. rapa* and *B. juncea* genetic resources. Data from FAO-WIEWS indicate that Australia, Germany, Spain, UK and USA conserve more than 25 representative species of *Brassica* including wild types (**Figure 3B**). Although the FAO promotes sharing of plant genetic resources for food and agriculture (PGRFA) through its sustainable developmental goals (SDGs), much work has to be done to ensure that the currently available biodiversity of *Brassica* species is not lost. Crop wild types are excellent sources for breeding for development of disease tolerant and abiotic stress tolerant plants that would be suitable for fresh challenges posed by the changing climate conditions.

## 3 Conservation methods for *Brassica* germplasm

The Seed Information Database (SID) maintained by Millennium Seed Bank of the Royal Botanic Gardens, Kew, UK designate all the 48 taxa of *Brassica* (species and subspecies) stored by them to be orthodox seeds. The orthodox nature of seeds from *Brassica* has in fact been well established (Genesys, 2017; Solberg et al., 2020). Therefore, long-term storage at -18 to -20°C for 40-60 year range is possible (Solberg et al., 2020). This explains the nature of storage of *Brassica* seeds, where a majority of germplasms are currently held in long term storage (**Figure 2C**) (Weise et al., 2017). However, other types of storages such as *in vitro* collection, DNA collection, cryopreservation are also employed to preserve *Brassica* germplasm, but scarcely used. Further studies or research are warranted on conserved germplasms to not only understand the characteristics and traits of stored seeds, but also to improve the currently followed storage practices (Acker et al., 2017; Panis et al., 2020). Several studies have been conducted in the past on monitoring dormancy, re-generation and suitability/effects of different temperatures as well as periods of storage for varied plants (Ellis et al., 2018; van Treuren et al., 2018; Everingham et al., 2021). Germplasms of *Brassica* conserved in gene banks have also been subjected to studies to optimize their storage conditions, regulation of seed dormancy and elucidation of useful characteristics such as metabolite profiling.

Seeds from members of family *Brassicaceae*, have been reported to commonly have a half-life (P_50_) of 54 years and some plants such as cabbage (*Brassica oleracea* L.) can even be maintained at +20°C and 50% RH up to 5 years in paper bags (Nagel and Börner, 2010; Solberg et al., 2020). Seeds of *B. villosa* ssp. drepanensis exhibited a high germination rate (92%) when stored at -20°C with a moisture content of 3% to 6% for 16 years. However, room temperature stored seeds succumbed to rapid deterioration after 3 to 4 years (Scialabba et al., 2016). Study of long term (36 years) stored *Brassicaceae* members showed that desiccation to reduce seed water content to less than 2.5% of fresh weight significantly improved germination at the end of storage. This effect was enhanced by enrichment of the storage environment with carbon di oxide (CO_2_) (Gonzalez-Benito et al., 2011). Hermitic storage of 37 *Brassica*ceae members including *B. napus*, *B. fruticulosa* and *B. fruticulosa glaberrima* at −5°C to −10°C with seed moisture content of 0.3 to 3%, 20% RH, indicated sustenance of seed viability over a period of 38-40 years (Pérez-García et al., 2007). Similar studies on seeds of *Brassica fruticulosa* and *B. repanda* stored at −5°C to −10°C with seed moisture content of <3%, 20% RH, over a period of 40 years from 1966 to 2006 also indicated sustained viability, displaying a higher germination rate of over 70% without use of dormancy-breaking agents such as gibberellic acid (Pérez-García et al., 2009). A study comparing *Brassica rapa* L. ssp. *pekinensis* and *Capsicum annuum* L. long term (10 years) seed storage using different packaging material indicated that *B. rapa* L. ssp. *pekinensis* was the most affected by storage at ambient temperatures even when packaged in vacuum-sealed aluminum pouches, but could be successfully stored for up to 10 years at 5°C/RH 30, using vacuum-sealed aluminum pouches (Soh et al., 2014). From these studies it can be understood that while some *Brassica* plants such as Cabbage (*Brassica* oleracea L.) can be stored under ambient temperatures (+20°C) it may not be suitable for others plants such as *B. rapa* L. ssp. *pekinensis* and *B. villosa* ssp. *drepanensis* which are to be stored at much lower temperatures of -5°C to -20°C. Other optimal conditions reported for *Brassica* members include a relative humidity of less than 50%, moisture content of <6%, use of CO_2_ if stored in sealed containers or use of vacuum sealed aluminum pouches. Also, extreme desiccation of seeds during storage have been seen to have a detrimental effects on seed longevity on plants such as *B. repanda* (Mira et al., 2015).

Other studies on *Brassica* species conserved in gene banks are found to be on the biochemical changes that occur in seeds during long term storage and screening of collections for their metabolites. Studies on seed lipid peroxidation and membrane permeability in *Brassica*ceae members during long term storage and their effect on seed germination, vigor indicated that increased membrane permeability to occur in *Brassica repanda* seeds which led to reduced germination % as well as vigor loss (Mira et al., 2011). In another study, a seed lipid thermal fingerprinting using differential scanning calorimetry (DSC) was proposed, which used several biophysical markers to predict performance of oily seeds of *Brassica*ceae during long-term storage (8-44 years) (Mira et al., 2019). A study on 168 accessions of *Brassica rapa* on understanding relationships between genetic markers and metabolites developed a set of genetic markers could be used for rapid selection of specific metabolite producing *B. rapa* genotypes (Pino Del Carpio et al., 2011).

## 4 Challenges in *Brassica* germplasm conservation

### 4.1 Taxonomic predicament of *Brassica* germplasm in genebanks

A major concern with worldwide *Brassica* germplasm collections often is the uncertainty associated with the naming of plant genetic resources. Descriptions of the origins, phenotypic and genotypic diversity of several *Brassica* members have been published (Dixon, 2006; Bonnema et al., 2011). However, naming of the *Brassica* members hitherto remains complex and often ambiguous due to differences in the naming conventions. Proper phylogenetic organization of the members of *Brassica* has been arduous due to extensive crossing and hybridization over centuries which occurred across geographically isolated microenvironments causing difficulties in tracing and comparison (Bonnema et al., 2011; Fahey, 2016). There is also an overlap of plant forms and morphological traits across species which further complicates phenotypical phylogenetic assignment and organization (OECD, 2016). The origins of modern *Brassica* vegetables and their taxonomy continues to be unsettled and regarded to be in a state of constant flux (Fahey, 2016).

**Figure 4:**
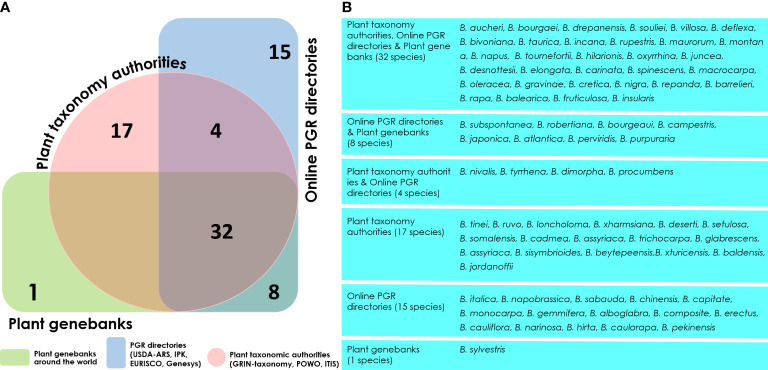
Venn diagram illustrating the distribution of *Brassica* species. **(A)** number of *Brassica* species available across Plant taxonomy authorities (Integrated Taxonomic Information System-ITIS, Plants of the world online-POWO, GRIN taxonomy), online plant genetic resource directories (WIEWS database, Genesys and EURISCO) and plant genebanks. **(B)** List of *Brassica* taxa in groups specified in the Venn diagram.

**Figure 5:**
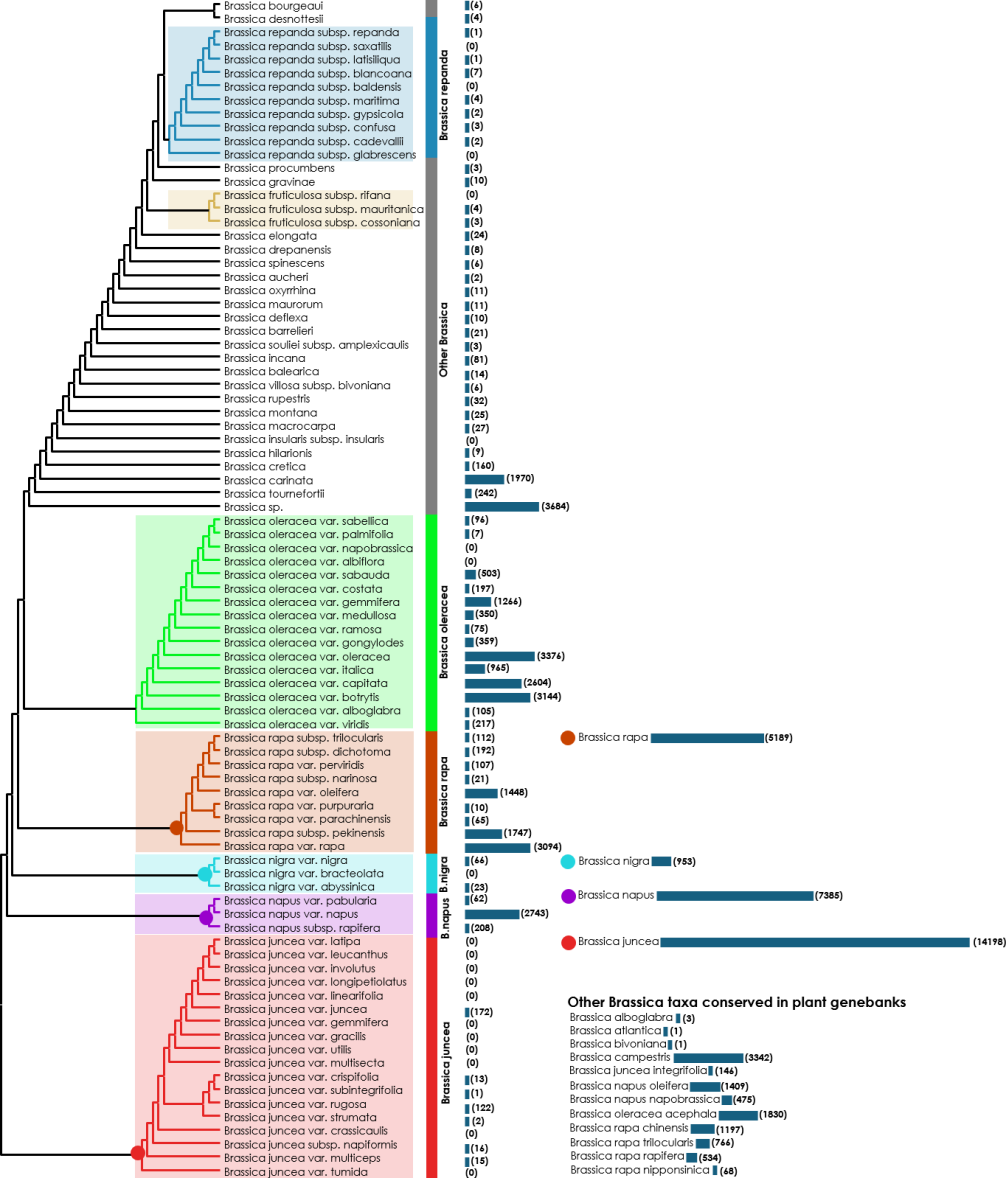
A common tree drawn using the NCBI taxonomy database (Sayers et al., 2019; Schoch et al., 2020). The base Newick’s tree was downloaded from the NCBI taxonomy database and redrawn using MEGA v. 11. Bars along with numbers in the parentheses indicate the number of accessions available according to the WIEWS database.

At present, only 13 subordinate taxa in the rank of species are recognized by the Integrated Taxonomic Information System (ITIS) in the genus *Brassica* (ITIS, 2022) (as on 05^th^ October, 2022) (**Table S2**). The ITIS is a taxonomic database maintained by the United States government which partners with other governmental agencies of Canada and Mexico as well as organizations dedicated to biodiversity such as GBIF (Global Biodiversity Information Facility) (GBIF, 2020). However, other well-recognized online catalogues as well as germplasm collections do not necessarily follow these naming conventions. For example, as on 05^th^ October, 2022, the Plants of the World Online-Royal Botanical Gardens, Kew in collaboration with World Flora Online project, provides taxonomic recognition of 41 species of *Brassica* (Borsch et al., 2020; POWO, 2022) (**Table S2**). These names are accepted and added to its ‘World Checklist of Seed Plants’ (Govaerts et al., 2022). Another repository BrassiBase, dedicated towards developing taxonomic, evolutionary, systematics and germplasm knowledge systems specifically for *Brassicaceae*, reports 45 species of *Brassica* (Kiefer et al., 2013) (**Table S2**). The GRIN-taxonomy resource which provides taxonomic information on plants conserved by the United States National Plant Germplasm System (USNPGS) remains as the only provider of taxonomic information along with conserving germplasm. The GRIN-taxonomy also currently recognizes 41 species of *Brassica*. But they also are not completely in agreement with the accepted list of *Brassica* species given the World Checklist of Seed plants. On a comparison of the *Brassica* taxa recognized by the plant taxonomy authorities, online PGR directories and plant genebanks we recognized issues related with the naming of *Brassica* taxa at species level (**Figure 4**). There are 18 *Brassica* species that are present only among the plant taxonomy lists (ITIS, POWO, BrassiBase & GRIN-Taxonomy) which are not conserved under the designated name at any germplasm conservation center or present in the list of *Brassica* species in the online repositories (Genesys & EURISCO) (**Figure 4A**). Similarly, it can also be observed that there are 15 *Brassica* species which are listed only in the online PGR directories (Genesys & EURISCO). This could be solved as they all of them are subspecies of *Brassica*, particularly *Brassica oleracea* and *B. rapa* which have been misnamed or updated to a new taxon (**Figure 4B**). Same is the case of *Brassica sylvestris* which was formerly designated as *Brassica rapa* subsp. *sylvestris* which is now recognized only in plant genebanks but not by plant taxonomy authorities or online PGR directories. The NCBI taxonomy database under *Brassica* (Taxonomy ID:3705) indicates 129 distinct taxa which after removing species crosses and taxa marked ambiguous names such as groups provide 87 unique taxa (**Figure 5**). These 87 unique taxa fail to include all the *Brassica* species conserved in plant genebanks.

The ambiguity of *Brassica* naming also continues to exist at the ground level. To begin with, the plant genetic resources storage centers around the world which are responsible for conservation and distribution of germplasm do not reflect the diverse species richness in *Brassica* germplasm conserved by them. The Gene bank Information System of the Leibniz Institute of Plant Genetics and Crop Plant Research (GBIS-IPK) indicates storing of accessions belonging to 31 species of *Brassica* whereas the United States Department of Agriculture- Germplasm Resource Information Network (USDA-GRIN) stores 24 different species of *Brassica* (excluding *Brassica* sp.) (Brassica-dataset, 2015; Oppermann et al., 2015) (**Table S2**). It is understandable that plant genebanks concentrate towards conserving local resources and rely on crop nomenclature. However, as most of them follow the *ex situ* model of conservation, they can also be used as an effective tool in protecting and documenting the *Brassica* biodiversity. For example, as several of the accepted taxa of *Brassica* such as *Brassica assyriaca, B. baldensis, B. beytepeensis* etc. have been officially added as verified and accepted species (Govaerts et al., 2022), plant genetic resource centers around the world can attempt to work towards taxonomic reclassification and updating of information on the bioresources which they conserve.

The problem of taxonomic uncertainties becomes much more complex across genebanks all around the world when describing the status at much lower taxonomic levels such as subspecies, cultivars and varieties which are often interchanged depending on the author/gene bank accessed. Such issues at times can complicate the taxonomic assignment after breeding or during research. Introduction of universal code for naming at this juncture, would be a tedious as well as an incredible task. A polymerase chain reaction (PCR) based approach for identification of *Brassica* species has been proposed albeit its efficacy is limited within the species from the U triangle (Koh et al., 2017). There is a possibility that this problem could be solved by help of genetic sequencing approaches. Since the advancement of sequencing technologies, genomic component based typing followed by distinctive universal taxonomic assignments have helped to overcome such barriers. This would also shorten the time required by traditional identification using phenotyping. The current requirement on *Brassica* taxonomy would therefore involve a polyphasic approach for phylogenetic organization which would combine more than one of the following: phenotyping or studying morphological traits; use of cytological markers, karyotyping; phytochemical, storage protein profiling; studying isozyme markers; microsatellite markers such as SSRs (short tandem repeats), SNPs (single nucleotide polymorphisms); and sequencing methods such as specific-locus amplified fragment sequencing (SLAF-seq) and using large scale genomic analyses and fingerprinting to categorize *Brassica* species (Pino Del Carpio et al., 2011; Liu et al., 2013; El-Esawi, 2017; Fotev et al., 2018; Yang et al., 2018; Bhandari et al., 2020; Chao et al., 2020; He et al., 2021; Rakshita et al., 2021; Hong et al., 2022; Li et al., 2022)

### 4.2 Diverse morphotypes and cytogenetics of *Brassica*

**Figure 6:**
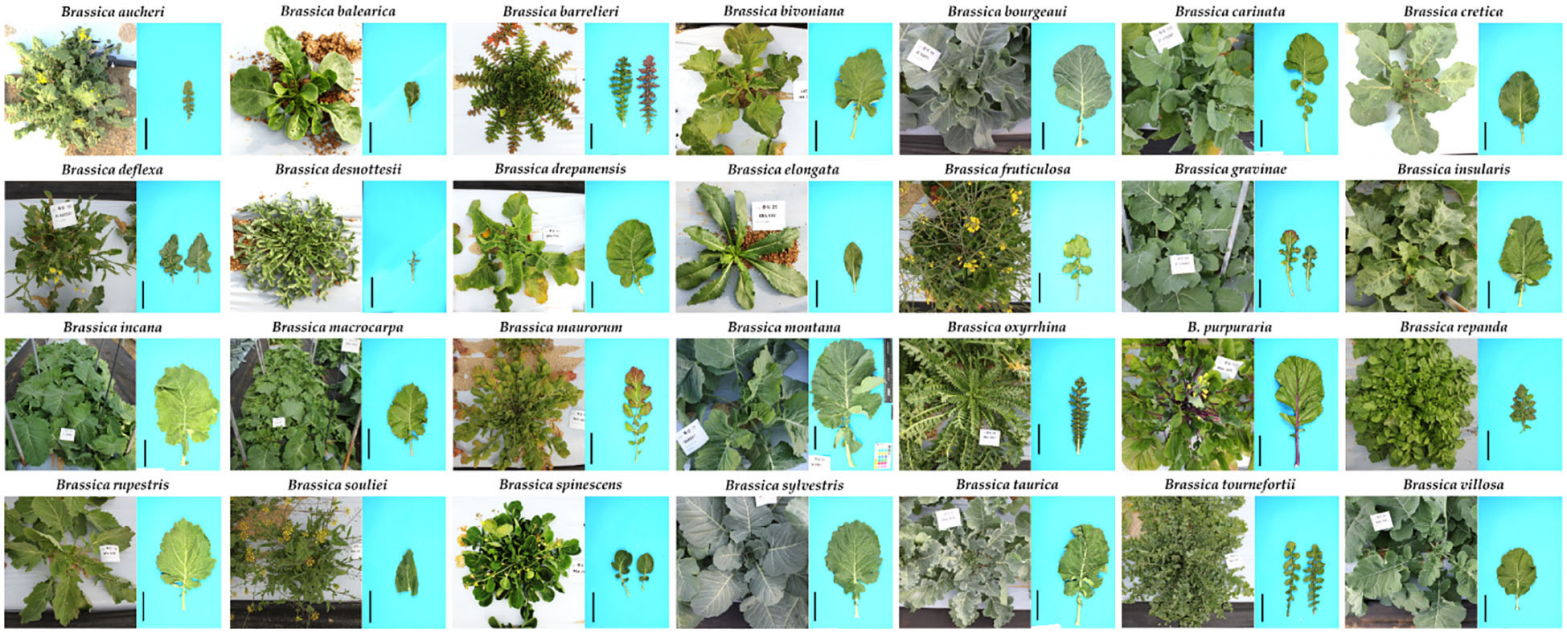
Morphologically varied wild type species of *Brassica*. The scale bars (in black) adjacent to the individual leaves represent a length of 10 cm and were included using ImageJ software (Unpublished data).

The richness of both species and morphotypes in *Brassica* can be understood to have arisen after the whole genome triplication (WGT) event that has been accepted (Arias et al., 2014; Cheng et al., 2014). Among the speciation concepts of *Brassica*, the U triangle is the most famous and accepted concept of cytogenetic relationships between the species belonging to the genus *Brassica* (**Figure S1**). The genome types of the ancestral diploid and tetraploid as explained in the triangle of U has been well discussed (Branca and Cartea, 2011). In addition to the six species of *Brassica* explained by the U triangle, other species of *Brassica* have also been continually described (Govaerts et al., 2022) (**Figure 6**). These are morphologically distinct compared to the six species and not much information is available about them for scientific research. There is a marked morphological diversity among various species of *Brassica* where some form heads or curd or stem and lead modifications. This variation among the species can be observed in their leaves – size, shape, margins, presence of anthocyanin; stems; habit; flower color and roots. To give few examples, the *Brassica* species *B. spinescens* and *B. balearica* form slight succulent leaves which is unique among various *Brassica* and *B. maurorum* produces minute flowers which look like an inflorescence (**Figure 6**). Some plants grow more than 2 meters in height (*B. rapa)* where are some (*B. elongata, B. balearica* and *B. maurorum*) do not grow more than 30 cm in height. Despite these plants having a long history of documentation and exhibiting significant difference in their morphology, habit and growth periods, they have not been studied in detail or conserved in large number in plant genebanks. In the recent decades, adequate emphasis has been given in literature to the importance of conservation of wild type species. These other *Brassica* species are vital as they can help to deal with the bottleneck problems associated with *Brassica* breeding which warrants the need for conservation of these wild type species of *Brassica*.

### 4.3 Technical hurdles in cultivation of *Brassica* species

Members of the family *Brassicaceae* family especially the vegetable crops, prefer to grow under cooler temperate conditions (15–20°C), with high humidity, but some species are also found to grow also under subtropical and even tropical zones (Fahey, 2016; Lin et al., 2018; Razzaq et al., 2021). For example Chinese cabbage plants grow well at a temperatures range of 13–20°C but prefer warmer growing conditions and often bolt at lower temperatures (Salehi et al., 2021). *Brassica juncea* or mustard plants widely grown on northern parts of India also prefer warmer temperatures as high as 27°C and can tolerate temperatures as low as 6°C (Shekhawat et al., 2012). Among other species, Brussels sprouts and kale are the most resistant to low temperatures, as they can tolerate temperatures as low as −12°C. In addition to preference of longer photoperiods for optimized growth, *Brassica* vegetables such as kohlrabi, Chinese cabbage, cabbage have a weaker root system and require humus rich or loamy soil with pH near neutral (6.2-7.0) and high water retention properties (Dhaliwal, 2017; Razzaq et al., 2021). Therefore, propagation of diverse *Brassica* species requires environment controllable facilities and not all of them can be multiplied in outdoor fields in all countries.

It has been reported that some *Brassica* species are self-fertile but most of them are self-incompatible and may require cross pollination to obtain seed (Stewart, 2002). This pollination can occur through wind, animals or insects or through human intervention. Though the human intervention aspects are related to breeding, cross-pollination by wind or insects such as bees are undesirable and can contaminate varieties. In case of wind pollination, isolation of plants is very helpful to avoid cross-pollination and incase of *B. napus* outcross could be reduced to less than 4% on separating plants by 120 cm; in oil seed rape almost 10% of pollen could be detected at a distance of even 360 m from the edge of the field; in radish also, the minimal distance for 0% crossing was at least 50 m (Stewart, 2002). The plant *B. napus* is highly prone to cross-pollination and spontaneous inter-specific hybridization of *B*. *napus* is possible with sexually compatible species (Meglic and Pipan, 2018). To produce pure seeds, *Brassica* plants need to be grown in isolation and cross-pollination risk reduces with the distance or introducing physical barriers in the flowering plants. However, as explained earlier, cross pollination is not a common phenomenon and rates are very low among *Brassica* spp. (Stewart, 2002). Avoidance of cross pollination can be carried by using a number of strategies including creating topographical barriers such as edges that would restrict or limit wind and insects, shifting of bee hives, establishment of border zones at the edges of the where seeds will not be collected from the plants in the zones and planting fields in such a way that the flowering time and period do not overlap (Stewart, 2002).

### 4.4 Diseases and pests

Almost all the *Brassica* vegetables are relatively susceptible to the same or similar diseases as well as insect pests (Fahey, 2016). Overall, pests are a serious threat to agricultural productivity and lately, with the exponential growth of *Brassica* cultivation, the distribution of *Brassica* vegetable pests have also increased (Witzel et al., 2021). The common microbial diseases occurring in *Brassica* species are tabulated (**Table S3**). The most common microbial infections in *Brassica* species are most caused by fungi especially *Leptosphaeria* (Blackleg disease), *Sclerotinia* (rot) and *Verticillium* (wilt). However, clubroot disease caused by *Plasmodiophora* is the highly reported disease among *Brassica* plants. Among bacterial pathogens *Pesudomonas* and *Pectobacterium* are the most common and Turnip yellow virus is the most common viral pathogen. In addition to diseases, *Brassica* crops are also attacked by a variety of pests that affect the growth as well as yield of *Brassica* members (**Table S4**). These causes a variety of issues ranging from feeding on the leaf tissues to killing the whole plants by feeding on plant roots. The most common insect pests of *Brassica* include aphids, worms, mites and weevils. However, there are also several beneficial microorganisms and insects that aid growth of *Brassica* species. These help the plants grow and prevent predators from feeding on the plants. Microorganisms including bacteria and fungi have been reported to help *Brassica* species by stimulating plant growth through mechanisms such as nitrogen fixation, siderophore production, alleviate chilling and heavy metal stress, and more importantly act as biocontrol agents against several bacterial and fungal diseases (Card et al., 2015). In addition to microbial interactions leading to enhanced growth of *Brassica* plants, some of the insects have also been reported to aid *Brassica* plants by acting as major predators of pests and feed on eggs of pests, aphids, small caterpillars, thrips and mites that attack the plants (**Table S5**).

## 5 Importance of conserving *Brassica* biodiversity

The culinary and therapeutic use of species in the U triangle has been well established and needs no emphasis. However, potential uses of other species are also currently gaining importance. For example, wild type accessions of *Brassica montana* and *B. balearica* were reported to exhibit resistance against the black rot caused by *Xanthomonas campestris* which particularly affects *B. oleracea* crops (Sheng et al., 2020). Similarly, *B. fruticulosa, B. hilarionis, B. macrocarpa, B. montana, B. spinescens* and *B. villosa* have been reported to exhibit antibiosis against the cabbage root fly (*Delia radicum*) which is a major threat to cabbage production in parts of Western Europe, the United States and Canada (Shuhang et al., 2016). Moderate resistance against other plant pathogen *Sclerotinia sclerotiorum* have reported in other *Brassica* species including *B. atlantica, B. bourgeai, B. incana, B. macrocarpa*, and *B. vilosa* (Taylor et al., 2018). Presence and applications of glucosinolates from *Brassica* has been well established in *B. oleracea, B. juncea* and *B. rapa.* The species *B. bourgeaui* has also been studied for their glucosinoates and have been reported to contain significantly high total glucosinotes than several *B. oleracea* crops in particular gluconapin, glucoraphanin and neoglucobrassicin (Tortosa et al., 2017). Glucosinolates have also been studied in *B. desnottesii, B. drepanensis, B. elongata*, and *B. gravinae* (Montaut et al., 2017; Malfa et al., 2022). All of the above studies have been made on germplasm obtained from plant genebanks and therefore, further research on the prospects of wild type *Brassica* can be significantly improved if these accessions are locally available for researchers.

## 6 Conclusions

The genus *Brassica* has given rise to a number of agriculturally important vegetable crops since ancient times. Moreover, it also provides the second major oilseed crop next only to soybean (Gupta, 2016). Conservation of *Brassica* germplasm and its biodiversity has still room for progress at the current scenario. *Brassica* vegetables has consistently been gaining popularity especially in the health food sector and are currently consumed across the Americas, Asia, and several European countries. Several parts of the plants are economically important depending on the species and those include leaves, seeds, oils. Thus their economic importance has multiplied and are now grown along with cereal crops to meet global demand (Salehi et al., 2021). With respect to medicine, members of genus *Brassica* are rich in biologically active metabolites which have been reported to possess potential health-promoting properties (Lee et al., 2013; Solov’yeva et al., 2013; Lee et al., 2014; Klopsch et al., 2017; Bhandari et al., 2020). Bioactive metabolites other than glucosinolates have also been identified and reported in *Brassica* which include alkaloids, anthocyanins, carotenoids, flavonoids, folates, other phenols, phytoalexins, phytosteroids and tocopherols (Pino Del Carpio et al., 2011; Branca et al., 2018; Lotti et al., 2018; Ramirez et al., 2020; Salehi et al., 2021). In addition to the health benefits of *Brassica* plants, in recent times, their commercial importance has grown in the food industry in which their morphological appeal to the consumers remains an important factor. This includes the appetizing appearance which is a combination of properties such as color, size, shape of the edible parts of the plant. Gene bank germplasm can therefore be screened for accessions with such attractive morphological traits. Earlier screening has been carried out for form/appearance significant *Brassica* vegetables including Kale and cauliflower (Thorwarth et al., 2018; Witzel et al., 2021). Further studies have also been made which included morphological properties of Chinese broccoli (*B. oleracea alboglabra*), and *B. rapa* (Fotev et al., 2018; Witzel et al., 2021). As crop wild relatives can serve as potential source of both biomedical compounds as well to improve commercial important phenotypic characteristics, conservation of the available biodiversity of *Brassica* becomes a prerequisite. In addition, they can also serve as a source of several useful traits which can be exploited to adapt crops to changing climate and improve biotic as well as abiotic resistances in *Brassica* crop species (Quezada-Martinez et al., 2021). Current status indicates conserved *Brassica* to be highly concentrated on few species and unevenly distributed across countries. Plant genebanks can provide an excellent solution to preserve the *Brassica* biodiversity for research, breeding, and commercial purposes.

## Author contributions

B-SH was involved in the conceptualization of the manuscript. PS and B-SH were involved in writing of the manuscript. S-HK was involved with data analysis, preparation of figures and tables. All authors contributed to the article and submitted and approved the submitted section.
