# Supplementary material for: Brassica biodiversity conservation: prevailing constraints and future avenues for sustainable distribution of plant genetic resources
Source: Front Plant Sci. 2023 Jul 27;14:1220134. doi: 10.3389/fpls.2023.1220134 (PMC10413119; doi:10.3389/fpls.2023.1220134)
Supplement: Supplementary file 1 [file DataSheet_1.docx]

Supplementary Material

Brassica Biodiversity Conservation: Prevailing Constraints and Future Avenues for Sustainable Distribution of Plant Genetic Resources

**Parthiban Subramanian^†^, Seong-Hoon Kim^†^ and Bum-Soo Hahn^*^**

*** Correspondence:** Corresponding Author: bshahn@korea.kr Supplementary Data

Table S1. *Ex situ* holdings of *Brassica* germplasm around the world (WIEWS, FAO) (as accessed on 1^st^ December, 2022)

| **Regions** | **Accessions** | **Species** | **Gene Banks** | **Countries** |
| --- | --- | --- | --- | --- |
| Eastern Africa | 2281 | 8 | 7 | 7 |
| Northern Africa | 329 | 10 | 4 | 4 |
| Southern Africa | 5 | 2 | 2 | 2 |
| Western Africa | 2 | 1 | 2 | 2 |
| **Africa** | **2,617** | **11** | **15** | **15** |
| Caribbean | 13 | 4 | 2 | 1 |
| Central America | 7 | 1 | 2 | 1 |
| Northern America | 6516 | 32 | 4 | 2 |
| South America | 1446 | 8 | 10 | 6 |
| **Americas** | **7,982** | **35** | **18** | **10** |
| Central Asia | 198 | 7 | 4 | 3 |
| Eastern Asia | 4240 | 10 | 2 | 2 |
| South-Eastern Asia | 1048 | 3 | 3 | 3 |
| Southern Asia | 18768 | 14 | 8 | 5 |
| Western Asia | 328 | 13 | 13 | 8 |
| **Asia** | **24,582** | **22** | **30** | **21** |
| Eastern Europe | 9156 | 22 | 20 | 9 |
| Northern Europe | 6221 | 28 | 10 | 5 |
| Southern Europe | 3874 | 34 | 24 | 10 |
| Western Europe | 7084 | 33 | 18 | 6 |
| **Europe** | **26,335** | **53** | **72** | **30** |
| Australia & New Zealand | 6637 | 26 | 3 | 2 |
| **Oceania** | **6,637** | **26** | **3** | **2** |
| ICBA^*^ | 200 | 2 | 1 | - |
| NORDGEN^*^ | 962 | 6 | 1 | - |
| SRGB^*^ | 1 | 1 | 1 | - |
| **Regional** | **1,163** | **6** | **3** | **-** |
| ILRI^*^ | 17 | 3 | 1 | - |
| ICARDA^*^ | 68 | 2 | 1 | - |
| WVC^*^ | 1977 | 6 | 1 | - |
| **International** | **2,062** | **6** | **3** | **-** |
| **TOTAL BRASSICA ACCESSIONS** | **71,378** |  | **144** | **78** |

* - Specific Genebanks: ICBA, International Center for Biosaline Agriculture; NORDEN, Nordic genetic resource center; SRGB, SADC Plant Genetic Resources Centre; ILRI, International Livestock Research Institute; ICARDA, International Centre for Agricultural Research in Dry Areas; WVC, world vegetable center

Table S2. Brassica species diversity published and accepted across various

| **Plant Taxonomic Authorities** | | | |  | **Online PGR catalogues** | |  | **Plant Genetic Resource Centers** | | | | |
| --- | --- | --- | --- | --- | --- | --- | --- | --- | --- | --- | --- | --- |
| **ITIS** | **POWO** | **BrassiBase** | **GRIN-taxonomy** |  | **Genesys** | **EURISCO** |  | **IPK** | **GRIN** | **UKVGB** | **RDA** |  |
| *B. carinata* | *B. assyriaca* | *B. aucheri* | *B. assyriaca* Mouterde |  | *Brassica sp.* | *Brassica sp.* |  | *Brassica sp.* | *Brassica sp.* | *Brassica sp.* | *Brassica sp.* |  |
| *B. cretica* | *B. aucheri* | *B. baldensis* | *B. aucheri* Boiss. |  | *B. campestris* | *B. alboglabra* |  | *B. balearica* | *B. aucheri* | *B. atlantica* | *B. balearica* |  |
| *B. elongata* | *B. baldensis* | *B. balearica* | *B. balearica* Pers. |  | *B. carinata* | *B. atlantica* |  | *B. barrelieri* | *B. balearica* | *B. balearica* | *B. barrelieri* |  |
| *B. fruticulosa* | *B. balearica* | *B. barrelieri* | *B. barrelieri (L.) Janka* |  | *B. cretica* | *B. balearica* |  | *B. bivoniana* | *B. barrelieri* | *B. carinata* | *B. campestris* |  |
| *B. juncea* | *B. barrelieri* | *B. beytepeensis* | *B. bourgeaui* (Webb ex Christ) Kuntze |  | *B. juncea* | *B. barrelieri* |  | *B. bourgeaui* | *B. bourgeaui* | *B. cretica* | *B. carinata* |  |
| *B. napus* | *B. beytepeensis* | ***B. bivoniana^*^*** | *B. cadmea* O. E. Schulz |  | *B. napus* | *B. bivoniana* |  | *B. carinata* | *B. carinata* | *B. hilarionis* | *B. deflexa* |  |
| *B. nigra* | *B. bourgeaui* | *B. bourgaei* | *B. carinata* A. Braun |  | *B. nigra* | *B. bourgeaui* |  | *B. desnottesii* | *B. cretica* | *B. incana* | *B. elongata* |  |
| *B. oleracea* | *B. cadmea* | *B. cadmea* | *B. cretica* Lam. |  | *B. oleracea* | *B. campestris* |  | *B. drepanensis* | *B. deflexa* | *B. insularis* | *B. fruticulosa* |  |
| *B. rapa* | *B. carinata* | *B. carinata* | *B. deflexa* Boiss. |  | *B. rapa* | *B. capitata* |  | *B. elongata* | *B. drepanensis* | *B. japonica* | *B. gravinae* |  |
| *B. rupestris* | *B. cretica* | *B. cretica* | *B. deserti* Danin & Hedge |  | *B. tournefortii* | *B. carinata* |  | *B. fruticulosa* | *B. fruticulosa* | *B. juncea* | *B. incana* |  |
| ***B. ruvo^*^*** | *B. deflexa* | *B. deflexa* | *B. desnottesii* Emb. & Maire |  | *Other* | *B. cauliflora* |  | *B. gravinae* | *B. gravinae* | *B. macrocarpa* | *B. juncea* |  |
| ***B. sisymbrioides^*^*** | *B. deserti* | *B. deserti* | *B. dimorpha* Coss. & Durieu |  |  | *B. caulorapa* |  | *B. incana* | *B. incana* | *B. montana* | *B. maurorum* |  |
| *B. tournefortii* | *B. desnottesii* | *B. desnottesii* | *B. drepanensis* (Caruel) Damanti |  |  | *B. chinensis* |  | *B. insularis* | *B. insularis* | *B. napus* | *B. montana* |  |
|  | *B. dimorpha* | *B. dimorpha* | *B. elongata* Ehrh. |  |  | *B. composita* |  | *B. juncea* | *B. juncea* | *B. nigra* | *B. napus* |  |
|  | *B. elongata* | *B. drepanensis* | *B. fruticulosa* Cirillo |  |  | *B. cretica* |  | *B. macrocarpa* | *B. macrocarpa* | *B. oleracea* | *B. nigra* |  |
|  | *B. fruticulosa* | *B. elongata* | *B. gravinae* Ten. |  |  | *B. deflexa* |  | *B. maurorum* | *B. maurorum* | *B. perviridis* | *B. oleracea* |  |
|  | *B. gravinae* | *B. fruticulosa* | ***B. xharmsiana* O. E. Schulz*^*^*** |  |  | *B. desnottesii* |  | *B. montana* | *B. napus* | *B. rapa* | *B. oxyrrhina* |  |
|  | *B. hilarionis* | ***B. glabrescens^*^*** | *B. hilarionis* Post |  |  | *B. dimorpha* |  | *B. napus* | *B. nigra* | *B. robertiana* | *B. rapa* |  |
|  | *B. incana* | *B. gravinae* | *B. incana* Ten. |  |  | *B. drepanensis* |  | *B. nigra* | *B. oleracea* | *B. rupestris* | *B. repanda* |  |
|  | *B. insularis* | *B. hilarionis* | *B. insularis* Moris |  |  | *B. elongata* |  | *B. oleracea* | *B. oxyrrhina* | *B. subspontanea* | *B. tournefortii* |  |
|  | *B. juncea* | *B. incana* | *B. juncea (L.)* Czern. |  |  | *B. erectus* |  | *B. oxyrrhina* | *B. rapa* | *B. sylvestris* |  |  |
|  | *B. loncholoma* | *B. insularis* | *B. loncholoma* Pomel |  |  | *B. fruticulosa* |  | *B. purpuraria* | *B. repanda* | *B. tournefortii* |  |  |
|  | *B. macrocarpa* | ***B. jordanoffii^*^*** | *B. macrocarpa* Guss. |  |  | *B. gemmifera* |  | *B. rapa* | *B. tournefortii* | *B. villosa* |  |  |
|  | *B. maurorum* | *B. juncea* | *B. maurorum* Durieu |  |  | *B. gravinae* |  | *B. repanda* | *B.villosa* |  |  |  |
|  | *B. montana* | *B. loncholoma* | *B. montana* Pourr. |  |  | *B. hilarionis* |  | *B. rupestris* |  |  |  |  |
|  | *B. napus* | *B. macrocarpa* | *B. napus* L. |  |  | *B. hirta* |  | *B. souliei* |  |  |  |  |
|  | *B. nivalis* | *B. maurorum* | *B. nigra* (L.) W. D. J. Koch |  |  | *B. incana* |  | *B. spinescens* |  |  |  |  |
|  | *B. oleracea* | *B. montana* | *B. nivalis* Boiss. & Heldr. |  |  | *B. insularis* |  | *B. sylvestris* |  |  |  |  |
|  | *B. oxyrrhina* | *B. napus* | *B. oleracea* L. |  |  | *B. italica* |  | *B. taurica* |  |  |  |  |
|  | *B. procumbens* | *B. nigra* | *B. oxyrrhina* (Coss.) Willk. |  |  | *B. japonica* |  | *B. tournefortii* | |  |  |  |
|  | *B. rapa* | *B. nivalis* | *B. procumbens* (Poir.) O. E. Schulz |  |  | *B. juncea* |  | *B. villosa* |  |  |  |  |
|  | *B. repanda* | *B. oleracea* | *B. rapa* L. |  |  | *B. macrocarpa* |  |  |  |  |  |  |
|  | *B. rupestris* | *B. oxyrrhina* | *B. repanda* (Willd.) DC. |  |  | *B. maurorum* |  |  |  |  |  |  |
|  | *B. setulosa* | *B. procumbens* | *B. rupestris* Raf*.* |  |  | *B. monocarpa* |  |  |  |  |  |  |
|  | *B. somalensis* | *B. rapa* | *B. setulosa* (Boiss. & Reut.) Coss. |  |  | *B. montana* |  |  |  |  |  |  |
|  | *B. souliei* | *B. repanda* | *B. souliei* (Batt.) Batt. |  |  | *B. napobrassica* |  |  |  |  |  |  |
|  | *B. spinescens* | *B. rupestris* | *B. spinescens* Pomel |  |  | *B. napus* |  |  |  |  |  |  |
|  | ***B. taurica^*^*** | *B. somalensis* | *B. spp.* |  |  | *B. narinosa* |  |  |  |  |  |  |
|  | *B. trichocarpa* | *B. souliei* | *B. tournefortii* Gouan |  |  | *B. nigra* |  |  |  |  |  |  |
|  | *B. tyrrhena* | *B. spinescens* | ***B. xturicensis* O. E. Schulz & Thell. *^*^*** |  |  | *B. nivalis* |  |  |  |  |  |  |
|  | *B.villosa* | ***B. tinei^*^*** | *B. villosa* Biv. |  |  | *B. oleracea* |  |  |  |  |  |  |
|  |  | *B. tournefortii* |  |  |  | *B. oxyrrhina* |  |  |  |  |  |  |
|  |  | *B. trichocarpa* |  |  |  | *B. pekinensis* |  |  |  |  |  |  |
|  |  | *B. tyrrhena* |  |  |  | *B. perviridis* |  |  |  |  |  |  |
|  |  | *B.villosa* |  |  |  | *B. procumbens* |  |  |  |  |  |  |
|  |  |  |  |  |  | *B. purpuraria* |  |  |  |  |  |  |
|  |  |  |  |  |  | *B. rapa* |  |  |  |  |  |  |
|  |  |  |  |  |  | *B. repanda* |  |  |  |  |  |  |
|  |  |  |  |  |  | *B. robertiana* |  |  |  |  |  |  |
|  |  |  |  |  |  | *B. rupestris* |  |  |  |  |  |  |
|  |  |  |  |  |  | *B. sabauda* |  |  |  |  |  |  |
|  |  |  |  |  |  | *B. souliei* |  |  |  |  |  |  |
|  |  |  |  |  |  | *B. spinescens* |  |  |  |  |  |  |
|  |  |  |  |  |  | *B. subspontanea* |  |  |  |  |  |  |
|  |  |  |  |  |  | *B. taurica* |  |  |  |  |  |  |
|  |  |  |  |  |  | *B. tournefortii* |  |  |  |  |  |  |
|  |  |  |  |  |  | *B. tyrrhena* |  |  |  |  |  |  |
|  |  |  |  |  |  | *B.villosa* |  |  |  |  |  |  |
|  | | | | | | | | | | | |  |

***** & bold indicate unique names only found in that specific database. ITIS: Integrated Taxonomic Information System; POWO: Plants of the world online; IPK: Institut Für Pflanzengenetik Und Kulturpflanzenforschung; GRIN: Germplasm Resources Information Network; UKVGB: UK vegetable database; RDA: Rural Development Administration, S. Korea.

Table S3. Common microbial diseases affecting Brassica species. (Source: Publications on microbial diseases of *Brassica* spp. during 2010-2022 & first reports published in the journal “Plant Disease”)

| **Plant** | **Disease** | **Microbial Pathogen** |
| --- | --- | --- |
| Brassica greens | Bacterial leaf spot | *Pseudomonas syringae pv. maculicola, Xanthomonas campestris pv. campestris* |
| Brocolli | Powdery Mildew, Bacterial leaf blight, Head rot, Basal drop and white mold, Blackleg Disease, Leaf spot disease, Crown rot | *Erysiphe cruciferarum, Pythium polymastum, Pseudomonas syringae pv. alisalensis, Fusarium tricinctum, Sclerotinia sclerotiorum, Leptosphaeria biglobosa, Alternaria brassicicola, Rhizoctonia solani* |
| Brussel sprouts | Bacterial Blight | *Pseudomonas cannabina pv. alisalensis* |
| Cabbage | Bacterial leaf blight, Blackleg Disease, Bacterial Blight, Soft rot, Foliar infection, Head rot, White mold, Root rot, Leaf spot disease, Turnip yellow virus, Fusarium wilt | *Plasmodiophora brassicae, Botryotinia fuckeliana, Aster Yellows Phytoplasma, Cabbage leaf curl virus, Pseudomonas syringae pv. alisalensis, Leptosphaeria biglobosa, Pseudomonas cannabina pv. alisalensis, Pectobacterium carotovorum subsp. Carotovorum, Phytophthora drechsleri, Pectobacterium wasabiae, Fusarium avenaceum, Sclerotinia sclerotiorum, Sclerotinia sclerotiorum, Pythium brassicum, Epicoccum sorghinum; Turnip yellow virus, Fusarium equiseti, Pectobacterium wasabiae* |
| Canola | Blackleg Disease, Powdery Mildew, Gray leaf spot, Damping-off, Charcoal rot, Sclerotinia Stem Rot, Clubroot, Phoma leaf spot | *Leptosphaeria biglobosa, Leptosphaeria maculans, Erysiphe polygoni, Alternaria brassicae, Phoma lingam, Rhizoctonia solani, Macrophomina phaseolina, Sclerotinia minor, Sclerotinia sclerotiorum, Rhizoctonia solani, Ceratobasidium sp., Plasmodiophora brassicae* |
| Cauliflower | Bacterial Blight, Root rot, Vascular wilt, Phyllody and flat stem | *Pythium polymastum, Xanthomonas campestris pv. campestris, Pseudomonas syringae pv. alisalensis, Pythium coloratum, Fusarium proliferatum, Candidatus Phytoplasma cynodontis* |
| Celery cabbage |  | *Cucumber mosaic virus* |
| Chinese cabbage | Damping-off, Powdery Mildew, Soft rot, Bacterial leaf spot, Bacterial leaf blight | *Alternaria japonica, Erysiphe cruciferarum, Broad bean wilt virus 2 family, Pectobacterium aroidearum, Pseudomonas viridiflava, Pantoea ananatis, Pantoea agglomerans, Verticillium dahliae* |
| Mustard | Basal drop and white mold | *Sclerotinia sclerotiorum* |
| Ethiopian mustard | Turnip mosaic disease, Sclerotinia Stem Rot, Powdery Mildew, Alternaria black spot | Turnip mosaic virus*, Sclerotinia sclerotiorum, Erysiphe cruciferarum, Alternaria alternata, Xanthomonas campestris pv. campestris* |
| Field mustard | Turnip ringspot, Blackleg Disease, Soft rot | Alternaria brassicae, Botrytis cinerea, Candidatus Phytoplasma asteris, Leptosphaeria biglobosa, Pectobacterium brasiliense,Pectobacterium carotovorum, Sclerotinia minor, Fusarium oxysporum, Turnip mosaic virus |
| Garlic mustard |  | *White clover mosaic virus, Turnip mosaic virus, Xanthomonas campestris* |
| Indian mustard | Powdery Mildew, White leaf spot, Powdery Mildew, Bacterial leaf blight, Blackleg Disease, Rot disease, Clubroot | *Erysiphe cruciferarum, Pseudocercosporella capsellae, Pseudomonas cannabina pv. alisalensis, Erysiphe cruciferarum, Leptosphaeria biglobosa, Rhizopus microsporus var. chinensis, Plasmodiophora brassicae* |
| Kale | Bacterial leaf spot, Black spot, Blackleg Disease, Soft rot, Root rot | *Xanthomonas campestris pv. campestris, Pseudomonas syringae pv. Tomato, Pectobacterium carotovorum subsp. brasiliensis, Leptosphaeria biglobosa, Rhizoctonia solani, Alternaria japonica* |
| Chinese Kale | Stem and root rot | *Fusarium incarnatum-equiseti Species Complex* |
| Siberian kale | Downy mildew | *Hyaloperonospora parasitica* |
| Oilseed rape | Phyllody, Black rot, Blackleg Disease | *Candidatus Phytoplasma asteris, Xanthomonas campestris pv. campestris, Turnip yellows virus, Leptosphaeria biglobosa* |
| Rapeseed | Powdery Mildew, Stem canker, Blackleg, Stem rot, Clubroot | *Erysiphe cruciferarum, Ralstonia solanacearum, Neopseudocercosporella capsellae, Alternaria brassicae, Verticillium longisporum, Pyrenopeziza brassicae, Plasmodiophora brassicae, Leptosphaeria maculans, Xanthomonas campestris pv. campestris, Sclerotinia sclerotiorum, Fusarium oxysporum, Turnip yellows virus, Turnip mosaic virus* |
| Turnip rape | Stem rot | *Sclerotinia subarctica nom. prov.* |
| Bok choy | Anthracnose, Soft rot | *Colletotrichum capsici, Dickeya dadantii subsp. dieffenbachiae, Pectobacterium brasiliense* |
| Rutabaga | Bacterial Blight | *Pseudomonas syringae pv. alisalensis* |

Table S4. Common pests that attack Brassica species. source: (Ekman et al., 2014)

| **Pest** | **Scientific name** | **Damage** |
| --- | --- | --- |
| African black beetle | *Heteronychus arator* | Larvae feed on plant roots leading to death. Adults can cause major damage by chewing the bases of plants and ringbarking seedlings. |
| Aphid – cabbage | *Brevicoryne brassicae* | Form large colonies on the youngest leaves which become stunted and distorted and spread cauliflower mosaic virus to brassicas. |
| Aphid – green peach | *Myzus persicae* | Causes leaf distortion through feeding, contaminates the product and potentially acts as a vector for many viruses. |
| Aphid – turnip | *Lipaphis erysimi* | Infest flowers and the undersides of leaves (particularly older leaves) causing them to become curled and yellow. Plant growth may be stunted. Spread viruses. |
| Cabbage centre grub | *Hellula hydralis* | Caterpillars feed on new growth, producing webbing as well as frass. Leaves can be webbed together. |
| Cabbage cluster caterpillar | *Crocidolomia pavonana* | Completely skeletonise leaves, depositing large amounts of webbing and frass as they grow. |
| Cabbage white butterfly | *Pieris rapae* | Large, irregular chewing damage to leaves and leaf edges, dark green droppings on leaves and in leaf angles. |
| Cluster caterpillar / Tropical armyworm | *Spodoptera litura* | Caterpillars skeletonise leaves. |
| Cutworm | *Agrotis spp.* | Larvae cut off seedlings at soil level |
| Diamondback moth | *Plutella xylostella* | Caterpillars fed on leaves and leave large holes, especially between leaf veins. |
| False wireworm / Vegetable beetle | *Gonocephalum spp.* | Larvae live in the soil where they feed on newly germinated seeds and plant roots. Adults chew stems at ground level, and may ring-bark small plants. |
| Flea beetle | *Phyllotreta spp.* | Adults feed on the plant leaves, causing small round pits or holes, while larvae feed on the plant roots. |
| Fungus gnats | *Bradysia spp.* | Larvae live near the soil surface where they feed on seedling roots and act as vectors for fungal diseases. |
| Green mirid | *Creontiades dilutus* | Adults and nymphs inject digestive enzymes into plants during feeding killing growing points. |
| Green vegetable bug | *Nezara viridula* | Young shoots are damaged by sap sucking. |
| Heliothis / Native budworm | *Helicoverpa armigera, H. punctigera* | Large, ragged holes in leaves, frass is a contamination issue. |
| Leafhopper / Jassid | *Family Cicadellidae* | All lifestages suck plant sap, reducing vigour and leaving whitish patches on the leaves. |
| Leafminer | *Liriomyza brassicae* | Females puncture leaves multiple times before laying an egg, causing leaf spots. Developing larvae make feeding tunnels inside the leaves. |
| Looper | *Chrysodeixis spp.* | Holes in leaves, leaves can be skeletonised. |
| Mite – blue oat | *Penthaleus spp.* | Active during cooler part of the day when it leaves the soil to feed on young leaves and shoots. Feeding damages the leaf surface, causing large whitish patches. |
| Mite – redlegged earth | *Halotydeus destructor* | Tears plant leaves to release sap, causing whitish patches on leaves. Mainly feeds in the morning or in overcast conditions. If disturbed it will drop to the ground and hide. |
| Mite – two spotted | *Tetranychus urticae* | Mites form colonies on lower leaf surfaces, especially near the petiole. These areas become covered in fine webbing. Feeding causes silvery speckling on the leaf surface and the leaves to become twisted and distorted. |
| Onion maggot / Seedcorn maggot | *Delia platura* | Larvae burrow into seeds and seedlings, destroying the seeds and stunting young plants. Cauliflower curds can be attacked, producing brown feeding trails. |
| Root knot nematode | *Meloidogyne spp.* | Swellings, knots and galls develop on infested roots. Upper parts of the plants may appear stunted, yellow and wilt easily.gs, knots and galls |
| Rutherglen bug | *Nysius vinitor* | Can cause some feeding damage through sap sucking, although vegetable crops are not preferred hosts. Main issue is contamination of fresh cut products. |
| Silverleaf whitefly | *Bemisia tabaci* | Larvae and adults suck sap from plants, stunting growth and reducing yield. Leaves develop silvery patches and may drop, while broccoli stalks can be bleached. Whiteflies excrete sticky honeydew, which encourages sooty mould growth, and can transmit some viruses. |
| Springtails | *Family Collembola* | Springtails feed on plant roots, causing extensive scarring. Roots can become brown or blackened, the plant wilts and may die. |
| Staphylinid beetle | *Family Staphilinidae* | The beetles feed on cauliflower curds, making them brown and unmarketable. |
| Thrips – onion, western flower | *Thrips tabaci, Frankliniella schultzei* | Feeding causes silvering of the leaves, in severe cases leading to leaf curling. The major damage potentially caused by certain thrips species is theirtransmission and spread of viruses (eg tomato spotted wilt virus) into the crop. |
| Weevil – apple | *Otiorhynchus cribricollis* | Larvae kill seedlings and young transplants by chewing through stems just below the soil surface. Adult weevils are nocturnal, spending the day burrowed shallowly into the soil. They emerge at night to feed on leaves, particularly the growing tips, and can ringbark young stems. |
| Weevil – small lucerne | *Atrichonotus taeniatulus* | Larvae kill seedlings and young transplants by chewing through stems just below the soil surface. Adult weevils feed on leaves and can ringbark young stems. |
| Weevil – spotted vegetable | *Desiantha diversipes* | Larvae kill seedlings and young transplants by chewing through stems just below the soil surface. Adult weevils feed on emerging leaves and can ringbark young stems. |
| Weevil – vegetable | *Listroderes difficilis* | Larvae and adults chew distinctive rounded holes in leaves during the evening and at night. Usually minor pest, damage is generally superficial but affects plant appearance and saleability. Heavy infestations can kill seedlings. |
| Weevil – white fringed | *Naupactus leucoloma* | Larvae live 5–15 cm deep in the ground where they eat plant roots. They can kill seedlings and young transplants by chewing through stems just below the soil surface. Adults feed on lower leaves but rarely cause major damage. |
| Wireworm | *Family Elateridae* | Larvae live in the soil where they feed on plant roots. |

Table S5. Beneficial insects that have been reported to help *Brassica* spp.

| Insect | Scientific name | Beneficial activity |
| --- | --- | --- |
| Damsel bug | *Nabis spp.* | Damsel bug nymphs and adults are extremely aggressive predators on other insects, including aphids, leafhoppers and various caterpillar species. |
| Ground beetle | *Carabidae spp.* | Larvae and adult beetles are predatory on insects, caterpillars, slugs, snails and other pests. They usually forage in soil litter or close to the ground. |
| Hoverfly | *Syrphidae spp.* | Maggots eat large numbers of aphids. However, larvae can present a contamination issue. |
| Lacewing – brown | *Micromus tasmaniae* | Adults and nymphs are voracious predators of aphids, small caterpillars, thrips and mites. However, can be a contamination issue, especially as the adults’ large wings may stick to wet leaves. |
| Lacewing – green | *Mallada signatus* | Adults and nymphs are voracious predators of aphids, small caterpillars, thrips and mites. |
| Ladybird – predatory | *Coccinella transversa, Hippodamia variegata, Diomus notescens* | Both adults and larvae are active predators of aphids, thrips, moth eggs and mites. However, ladybird larvae can represent a contamination risk. |
| Mite – predatory | *Phytoseiulis persimilis* | Predatory on two spotted mite and bean spider mite. |
| Parasitoid wasps | *Trichogramma spp., Telenomus spp., Diadegma spp.* | The adult females lay their eggs inside eggs, caterpillar or pupae of pest moths or butterflies, or directly into aphid adults or nymphs. One or many larvae live inside their host, eventually killing it. |
| Plague soldier beetle | *Chauliognathus lugubris* | Predatory on aphids, caterpillar eggs and other pests, which are supplemented with nectar and pollen. However, can be a significant contaminant issue. |
| Rove beetle | *Paederus spp.* | Predatory on various small insects. However, beetles contain a toxin which is released if they are damaged or crushed. This can cause extreme skin irritation, known as *Paederus dermatitus.* |


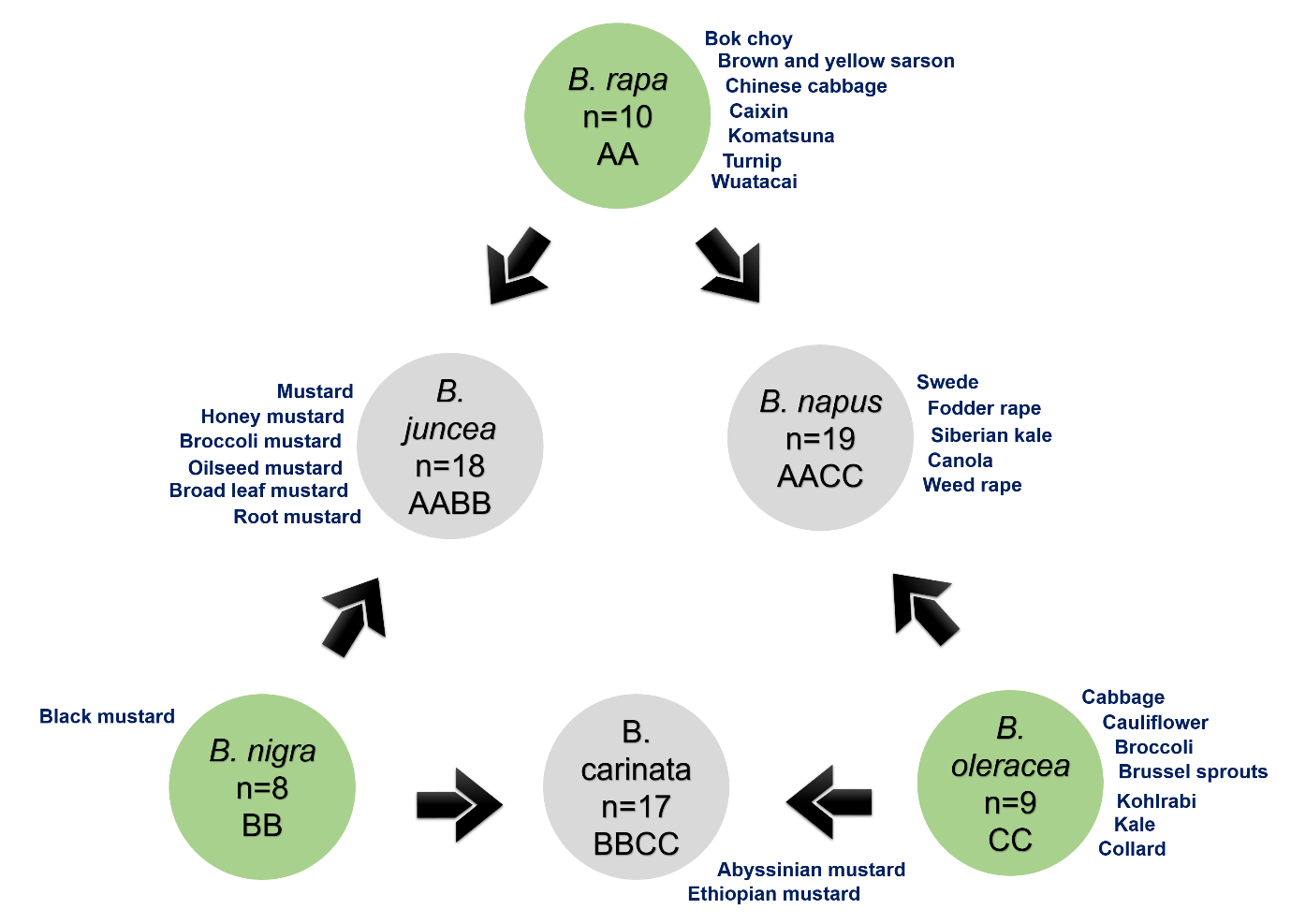


Figure S1. The speciation of Brassica with the aid of U’s triangle. Common names of the species are given adjacent to their scientific names.


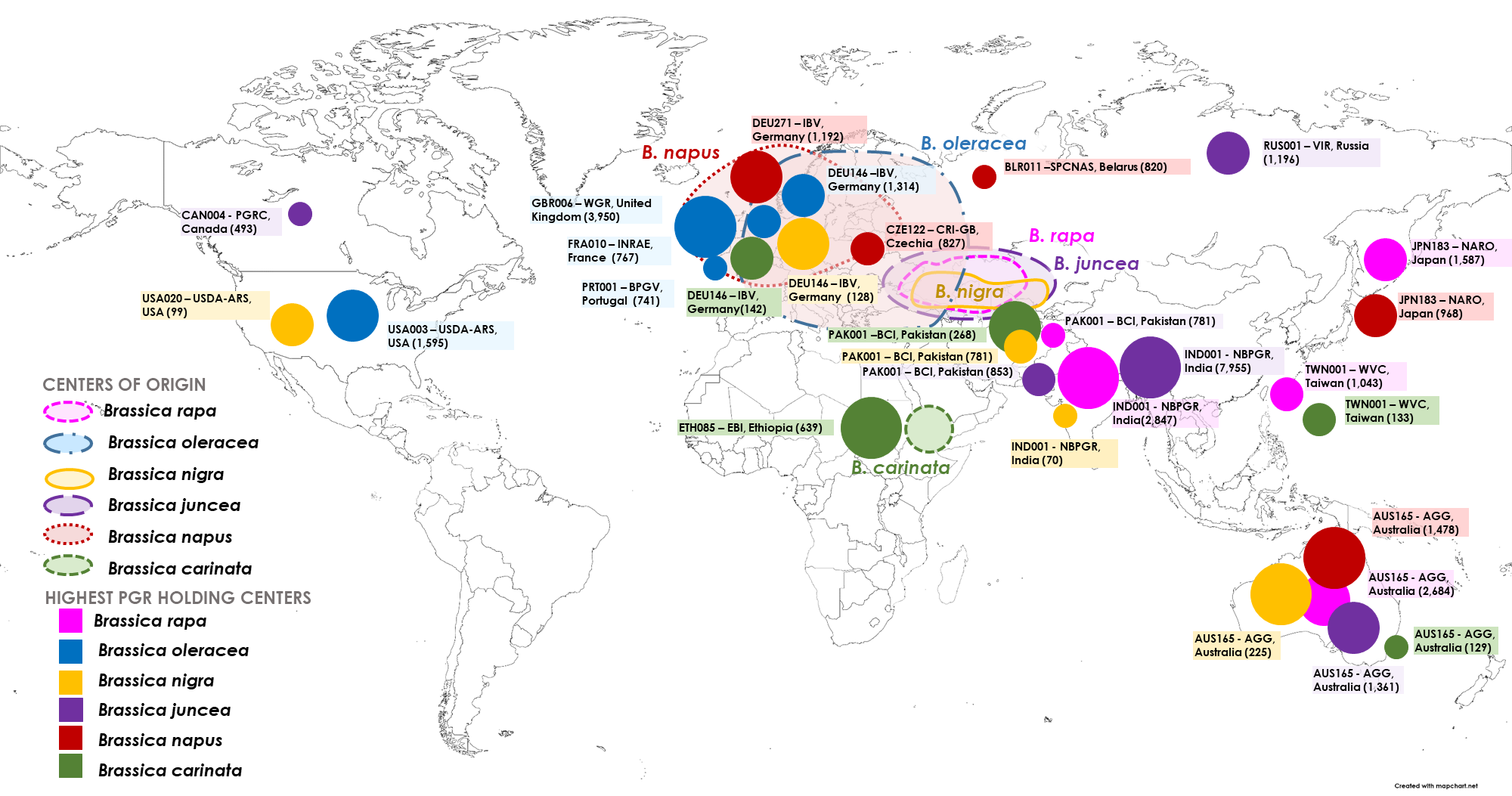


Figure S2. Origin of the six basic (diploid and tetraploid) species of Brassica and top five plant genetic resource centers holding highest number of each species. The labels contain the WIEWS institutional code (INSTCODE), name of organization and number of accessions held. AGG – Australian Grains Genebank; BCI – Bio-resources Conservation Institute; BPGV - Banco Português de Germoplasma Vegetal; CRI-GB - Crop Research Institute Gene bank; EBI - Ethiopian Biodiversity Institute; IBV - Information and Coordination Centre for Biological Diversity; INRAE - Institut de Génétique Environnement et Protection des Plantes, Plant Biology and Breeding; NARO - National Agriculture and Food Research Organization; NBPGR – National Bureau of Plant Genetic Resources; PGRC - Plant Gene Resources of Canada; SPCNAS - Scientific Practical Centre of the National Academy of Sciences of Belarus for Arable Farming; USDA-ARS – United States Department of Agriculture- Agricultural Research Service; VIR - N.I. Vavilov All-Russian Scientific Research Institute of Plant Industry; WGR - Warwick Genetic Resources Unit; WVC – World Vegetable Center.

**
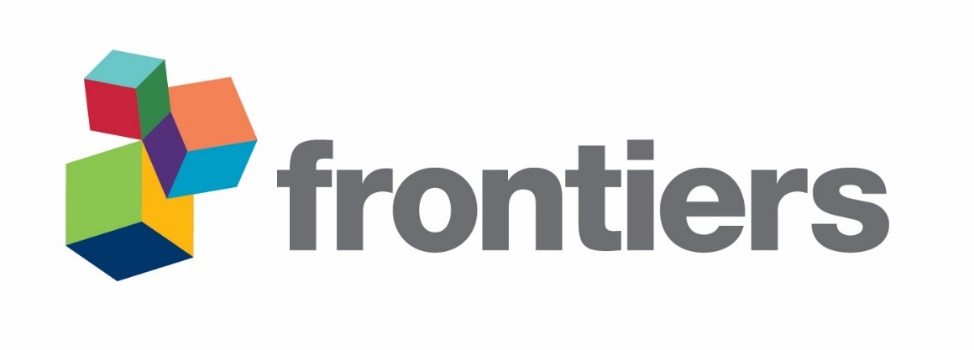
**

Ekman, J., Tesoriero, L., and Grigg, S. (2014). *Pests, diseases and disorders of brassica vegetables: a field identification guide.*
